# WTAP/IGF2BP3 mediated m6A modification of the EGR1/PTEN axis regulates the malignant phenotypes of endometrial cancer stem cells

**DOI:** 10.1186/s13046-024-03120-w

**Published:** 2024-07-23

**Authors:** Bo Wang, Yuting Wang, Wantong Wang, Zihao Wang, Yunzheng Zhang, Xin Pan, Xin Wen, Hongrui Leng, Jing Guo, Xiao-xin Ma

**Affiliations:** Department of Obstetrics and Gynecology, Shengjing Hospital of China Medical University, 39 Huaxiang Road, Tiexi District, Shenyang City, Liaoning Province 110022 China

**Keywords:** Endometrial cancer, Cancer stem cells, N6-methyladenosine RNA modification (m6A), Chemotherapy drug resistance

## Abstract

Endometrial cancer (EC) stem cells (ECSCs) are pivotal in the oncogenesis, metastasis, immune escape, chemoresistance, and recurrence of EC. However, the specific mechanism of stem cell maintenance in EC cells (ECCs) has not been clarified. We found that WTAP and m6A levels decreased in both EC and ECSCs, and that knocking down WTAP promoted ECCs and ECSCs properties, including proliferation, invasion, migration, cisplatin resistance, and self-renewal. The downregulation of WTAP leads to a decrease in the m6A modification of EGR1 mRNA, and it is difficult for IGF2BP3, as an m6A reader, to recognize and bind to EGR1 mRNA that has lost m6A modification, resulting in a decrease in the stability of EGR1 mRNA. A decrease in the EGR1 level led to a decrease of in the expression tumor suppressor gene PTEN, resulting in deregulation and loss of cellular homeostasis and thereby fostering EC stem cell traits. Notably, the enforced overexpression of WTAP, EGR1, and PTEN inhibited the oncogenic effects of ECCs and ECSCs in vivo, and the combined overexpression of WTAP + EGR1 and EGR1 + PTEN further diminished the tumorigenic potential of these cells. Our findings revealed that the WTAP/EGR1/PTEN pathway is important regulator of EC stem cell maintenance, chemotherapeutic resistance, and tumorigenesis, suggesting a novel and promising therapeutic avenue for treating EC.

**Supplementary Information:**

The online version contains supplementary material available at 10.1186/s13046-024-03120-w.

## Introduction

Endometrial cancer (EC) is the most common gynecological malignancy in developed regions, and approximately 76,000 women die from this disease every year worldwide. Although the number of diagnoses has increased across all age groups, the number of cases of EC has doubled among women under the age of 40 years [1, 2]. Despite advancements in early detection and the integration of surgical, chemotherapeutic, and immunotherapeutic strategies to increase patient survival rates, individuals with advanced or relapsed EC frequently experience suboptimal treatment outcomes and poor prognoses [2, 3]. Therefore, elucidating the mechanism underlying the occurrence and development of EC and identifying targeted therapies are highly important.

Cancer stem cells (CSCs) are a special subgroup of tumor cells that exhibit self-renewal, pluripotency, proliferation, metastasis, immune escape, and chemoresistance [4–6]. The identification and examination of cancer stem cells have substantially advanced the comprehension of and therapeutic approaches for treating malignancies. Some studies have shown that EC stem cells (ECSCs) can be extracted from EC cells (ECCs), estrogen-dependent Ishikawa cell line [7]. An increasing body of literature underscores the critical involvement of ECSCs in the progression of EC, identifying a pivotal area for future research and potential therapeutic intervention [8, 9].

In recent years, epigenetic mechanisms have been increasingly recognized for their pivotal role in the genesis and evolution of neoplasms [10]. N6-methyladenosine (m6A) is the most common post-transcriptional RNA modification in eukaryotic cells, and methyltransferase-like 3 (METTL3), methyltransferase-like 14 (METTL14) and Wilms’ tumor 1-associated protein (WTAP) together constitute the m6A methylase complex [11–13]. WTAP is instrumental in the nuclear localization of the m6A methylase complex, facilitating the recruitment of METTL3 and METTL14 [14, 15]. Demethylation mainly includes two demethylases, obesity associated protein (FTO) and alkB homologue 5 (ALKBH5) [16, 17]. WTAP plays an important role in m6A modification and has been shown to play an critical role in tumorigenesis and development [18, 19]. In recent years, more and more studies have shown that m6A modification plays an important role in CSCs [20].

Insulin-like growth factor 2 (IGF2) mRNA binding proteins (IGF2BPs) are a quintessential class of m6A readers that effectively identify m6A modifications and have a spectrum of regulatory functions pertinent to RNA [21]. The IGF2BP family includes IGF2BP1, IGF2BP2 and IGF2BP3 [21]. IGF2BPs generally play a role in stabilizing and storing mRNAs, thereby affecting gene expression and output [22].

Early growth response factor-1 (EGR1) regulates fibrogenic molecules, and aberrant expression of EGR1 leads to fibrosis [23]. EGR1 usually acts as a transcription factor and has a tumor suppressor function in various tumors, including breast cancer [24, 25]. However, the role of EGR1 in EC is still unclear and needs to be further explored.

Phosphatase and tensin homolog (PTEN), located on chromosome 10, is a formidable tumor suppressor [26]. The integrity of PTEN is crucial for the preservation of genomic stability, ensuring the equilibrium of cellular self-renewal processes and metabolic homeostasis [27, 28]. PTEN plays an important role in tumor regulation, and a small change in PTEN can affect tumor progression [29]. Recent studies have also shown that the loss of PTEN is involved in the maintenance of stemness in tumor stem cells [30]. PTEN has a classic anticancer role in EC, but its role in ECSCs has not been reported.

The sex determining region Y-box 2 (SOX2) has been identified in embryonic and adult stem cells [31, 32]. Recent research has revealed that SOX2 is a quintessential marker of cancer stem cells and is crucial for the preservation of their stemness [33]. Nanog homeobox transcription factor (NANOG) is pivotal for maintaining the stemness and pluripotency of cancer stem cells, as well as their self-renewal capabilities [34]. NANOG is also considered a marker of poor prognosis for varous tumors [35]. Both SOX2 and NANOG are critical markers of ECSCs [7]. In this study, we found that low expression of the m6A methylase WTAP reduced the m6A modification level on EGR1 mRNA, resulting in decreased binding between the m6A reader IGF2BP3 and EGR1 mRNA, thus reducing the stability of EGR1 mRNA. A decrease in the expression of EGR1, a transcription factor of PTEN, led to a decrease in the expression of the tumor suppressor gene PTEN, which increased the stemness of ECSCs; promoted tumor proliferation, invasion, migration, cisplatin resistance and self-renewal ; and inhibited the apoptosis of tumor cells.

## Materials and methods

### Human tissue specimens

A total of 50 EC tissues and 50 normal endometrial tissues were collected from patients undergoing surgery in the Department of Obstetrics and Gynecology at Shengjing Hospital of China Medical University from 2017 to 2020. Prior to surgery, these patients had not been subjected to either radiotherapy or chemotherapy treatments. The EC diagnoses were substantiated through histopathological examination of post-surgical paraffin-embedded tissue sections, adhering to the staging criteria set forth by the International Federation of Gynecology and Obstetrics (FIGO 2009). All patients were provided with informed consent. Informed consent was duly obtained from all participating patients. Ethical approval for this study was granted by the ethics committee of Shengjing Hospital at China Medical University, under the protocol number 2020PS270K.

### Bioinformatics analysis

We sourced RNA sequencing data (FPKM format) from the The Cancer Genomic Atlas (TCGA) database(portal.gdc.cancer.gov), with a specific focus on uterine corpus EC (UCEC). The dataset included RNA sequencing data from 23 UCEC tissues and 23 normal tissues paired with them. In addition, we used the UALCAN (ualcan.path.uab.edu) database to visualize RNA expression levels, which included 546UCEC tissues and 35 normal tissues from the TCGA. The GSE17025 dataset was downloaded from the GEO (www.ncbi.nlm.nih.gov/geo/) database and included 91 EC tissues and 12 normal tissues for RNA expression analysis. The ‘limma’ and ‘ggpubr’ packages are used in R (4.3.1) for data analysis and visualization. The“pRRophetic” packages in R (4.3.1) were used to evaluate the sensitivity of patients to chemotherapy drugs.

### RNA extraction and real-time fluorescence quantitative PCR

Total RNA was extracted from tissues or cells using Trizol reagent (Vazyme, Nanjing, China). The expression of target gene mRNA was detected by the 7500 fast real-time polymerase chain reaction (PCR) system (Applied Biosystems, USA) using a one-step SYBR primescript RT-PCR Kit (Vazyme, Nanjing, China). β-actin served as the endogenous reference gene for normalization purposes. The specific primer sequences employed within this research are delineated in Supplementary Table S1. Quantitative alterations in gene expression were determined via relative quantification method (2^^−ΔΔCt^).

### Cell lines and cell culture

The HEC-1B cells were purchased from the Shanghai Institute of Cell Biology at the Chinese Academy of Sciences (Shanghai, China). The HEC-1B cells were cultured in high glucose DMEM (Gibco, Carlsbad, CA, USA) supplemented with 10% fetal bovine serum (FBS; Gibco) and 1% penicillin-streptomycin (Invitrogen, Carlsbad, CA, USA).The Ishikawa human EC cell line was procured from the Department of Pathophysiology at Peking University (Beijing, China) in RPMI1640 medium (Gibco) supplemented with 10% fetal bovine serum (FBS; Gibco) and 1% penicillin-streptomycin (Invitrogen). From Ishikawa cell line, ECSCs were derived. The ECSCs were cultured in a serum-free medium (SFM) comprising a 1:1 mixture of DMEM/F12 (Gibco), supplemented with 1% penicillin-streptomycin (Invitrogen), HEPES (Amresco, Solon, USA), 5% bovine serum albumin (BSA) (Roche, Basel, Switzerland),2% B27, 1% Insulin Transferrin-Selenium, 20 ng/ml epidermal growth factor (EGF) and basic fibroblast growth factor (bFGF) (Peprotech, Rocky Hill, USA). The ECSCs were subsequently cultured in a 6-well low attachment surface plate provided by Corning (NY, USA) [5]. All cellular cultures were incubated at 37 °C in a humidified atmosphere containing 5% carbon dioxide.

### Cell transfection

Overexpressing viruses of WTAP, EGR1 and PTEN and their respective negative control (NC) counterparts were synthesized (GeneChem, Shanghai, China). ECSCs and Ishikawa cells were then transfected at a multiplicity of infection (MOI) of 20,

while an MOI of 10 was used for HEC-1B cells. The sequences for siRNAs targeting WTAP, EGR1, PTEN, IGF2BP1, IGF2BP2, and IGF2BP3, as well as their corresponding NC siRNAs, are detailed in Supplementary Table S2 (GenePharma, Shanghai, China). The transfection of cells with these siRNAs was performed utilizing Lipofectamine 3000, a reagent from Invitrogen, in strict adherence to the guidelines provided by the manufacturer.

### Flow cytometry

The cell spheres were induced and then isolated from the Ishikawa cells as described above. A single-cell suspension was prepared, utilizing 100 µl of buffer for every 10^7 cells, and subsequently stained with PE-CD133 and PE-Cy7-CD44 markers (BD, Franklin Lakes, NJ, USA). This staining process was conducted for 30 min in an environment shielded from light at a temperature of 4 °C, in preparation for cell sorting. The sorting was executed using the AriaIII system from BD Biosciences. The cells that were positive for both CD44 and CD133 were ECSCs (the cells that appeared in the upper right quadrant). At the same time, we backtested the selected stem cells and proved that most ECSCs were indeed positive for CD44 and CD133. The resultant population of cells, characterized by the co-expression of CD44 and CD133 markers, was then propagated in the stem cell media as delineated in the prior methodology.

### Sphere formation assay

After the ECSCs were isolated and enzymatically decomposed into discrete cellular entities, they were cultured for 7 days in a 6-well plate (Corning, NY, USA, REF3471) with a low attachment surface, strictly following the previously described ECSCs culture pattern. Subsequently, the spheroids were photographed with a microscope and their diameters were measured.

### Cell viability assay (CCK8 assay)

Cells were cultured within 96-well plates. Then 10 µL of CCK-8 (Cell Counting Kit-8; Dojindo, Japan) were added into per well. The plates were then incubated at 37 °C in an atmosphere containing 5% CO2 for a duration of 3 h, the OD450 value for each well was ascertained using a microplate reader (Bio-Rad, Hercules, CA, USA) at 0, 24 h, 48 h, and 72 h.

### Cell migration and invasion assay

Transwell chambers (Corning, NY, USA) featuring a pore size of 8 μm, were employed to assess cell migration and invasion capabilities. The cells were resuspended in 200µL of serum-free medium and seeded into the upper chamber of the Transwell apparatus. For invasion assays specifically, the upper chamber was pre-coated with Matrigel solution (BD, Franklin Lakes, NJ, USA). The lower chamber was filled with medium containing 10% FBS. Following a 24-hour incubation period, cells that had migrated or invaded through to the lower surface of the membrane were fixed using 4% paraformaldehyde and subsequently stained with 0.1% crystal violet solution. The enumeration of migrated and invaded cells was conducted across three randomly selected fields, with cell quantification facilitated by the ImageJ software.

### Chemosensitivity assay

Cells were subjected to treatments with varying concentrations of cisplatin, specifically 0.5, 1, 2, 4, 8, 16, 32, 64, and 128 µM, for a duration of 48 h. The CCK-8 assay was subsequently utilized to evaluate the impact on cell proliferation. Concentrations of 0.573 µM and 1.930 µM of cisplatin were chosen for Ishikawa and ECSCs, respectively, based on the half maximal inhibitory concentration (IC50) values determined from preliminary assays. The relative cell survival rate was calculated employing the formula: Relative Cell Survival Rate = (OD value of the cisplatin-treated group / OD value of the untreated group) × 100%).

### Apoptosis assay

Following transfection, we processed 1 × 10^6^ cells from each experimental group with a thorough wash in PBS. Subsequently, these cells were incubated at room temperature in a dark environment with phycoerythrin (PE) and fluorescein isothiocyanate (FITC) dyes for a duration of 15 min. To determine the percentage of apoptotic cells in the different experimental groups, we performed flow cytometry analysis. This quantification was carried out using a DxFLEX flow cytometer (Beckman, located in Suzhou, China).

### Western blot

Total proteins were extracted from either tissues or cells utilizing RIPA lysate (Beyotime, China, Catalogue P0013B). The protein samples were subsequently resolved via sodium dodecyl sulfate-polyacrylamide gel electrophoresis (SDS-PAGE) and transferred onto a polyvinylidene fluoride (PVDF) membrane. The membrane was then incubated in 5% skim milk for a duration of 2 h to block non-specific binding sites, followed by an overnight incubation at 4 ºC with the primary antibody. The following day, the membrane was incubated with horseradish peroxidase-conjugated secondary antibodies suitable for the primary antibodies used, this incubation lasting for 2 h at ambient temperature. Detection of the target proteins was achieved using an enhanced chemiluminescence (ECL) kit (Beyotime, China, Catalogue P0018AS), with the chemiluminescence signals captured and analyzed using Image Lab software (Beta 3.0, Bio-rad, California, USA). β-actin served as the internal control for normalization, and relative integral density values (IDV) were computed utilizing ImageJ software. The specific antibodies employed in this experiment are detailed in Supplementary Table S3.

### Immunohistochemistry

Paraffin-embedded samples, derived from either clinical specimens (endometrial carcinoma or normal endometrium) or xenograft tumors from nude mice, were selected for immunohistochemistry (IHC) analysis. Initially, the sections underwent a dewaxing process. Subsequently, they were immersed in Tris-Ethylenediaminetetraacetic acid (EDTA) buffer (pH 9.0, Solarbio, Beijing, China) and subjected to a heating process in a water bath at 100 °C for 10 min, followed by a re-equilibration to room temperature over a span of 30 min. The UltraSensitive™ SP (mouse or rabbit) IHC Kit (Maixin, Fuzhou, China, Catalogue kit-9710) was utilized to inhibit the endogenous peroxidase activity within the samples and to minimize non-specific staining. The development of the chromogenic reaction was facilitated by the 3,3’-diaminobenzidine (DAB) kit (Maixin, Fuzhou, China, Catalogue DAB-0031). Quantification of the stained areas was performed using an optical microscope. The specific antibodies employed throughout this procedure are delineated in Supplementary Table S3.

### RNA immunoprecipitation (RIP) assay

RIP was conducted utilizing the Magna RIP™ RNA-Binding Protein Immunoprecipitation Kit (Millipore, USA, Catalogue 17–700), strictly adhering to the provided manufacturer’s guidelines. A total of 5 µg of either WTAP, IGF2BP3, or FLAG antibody (Table S3) was employed, with IgG serving as the negative control (NC) and IgG as a NC. Initially, cell lysates were incubated with a concoction of RIP buffer, magnetic beads, and the designated antibodies to form a complex. This complex was subsequently treated with Proteinase K to facilitate the isolation of the immunoprecipitated RNA. The extracted RNA was then subjected to quantitative reverse transcription–PCR (RT-qPCR) analysis to validate the presence of the intended binding targets.

### RNA m6A quantification

Total RNA was extracted using the TRIzol reagent (Invitrogen, Carlsbad, CA, USA). Subsequently, the relative content of m6A modifications within the RNA was quantified using the EpiQuik m6A RNA Methylation Quantification kit (colorimetric) (EpiGentek, USA). The absorbance was ascertained at 450 nm utilizing a microplate reader, facilitating the determination of m6A levels.

### MeRIP-qPCR

MeRIP was conducted to examine m6A modification of genes using the Magna MeRIPTM m6A kit (EpiGentek, USA) according to the manufacturer’s instructions. Briefly, 10 µg aliquot of anti-m6A antibody (Synaptic Systems) was conjugated to ChIP grade protein A/G magnetic beads overnight at 4 °C. Three hundred microgram aliquot of fragmented total RNA was then incubated with the antibody in IP buffer with protease inhibitor and RNase inhibitor. The m6A modified RNA was then eluted with elution buffer, purified through Phenol/Chloroform/Isoamyl alcohol (25:24:1, Millipore) extraction, and then analyzed via quantitative RT-PCR assays. The primer sequences are shown in Supplementary Table S1.

### Chromatin immunoprecipitation (ChIP)

The ChIP assay was executed utilizing a kit (Active Motif, Shanghai, China), adhering closely to the provided manufacturer’s instructions. The procedure encompassed several key stages: initial cell cross-linking followed by ultrasonic fragmentation to break down the chromatin into manageable pieces. Subsequently, specific antibodies were introduced into the chromatin mixture: anti-EGR1 antibody to target the protein of interest, anti-RNA polymerase II serving as the positive control, and normal mouse IgG as the negative control, to ensure specificity of the assay. This mixture was then incubated overnight at a temperature of 4 °C to allow for adequate antibody-chromatin binding. Following the incubation period, the protein-DNA complexes were de-crosslinked to separate the DNA from the protein components. The DNA was then purified to remove any contaminants and enrich the sample for the sequences of interest. Primers were meticulously designed and synthesized, targeting the predicted binding sites within the promoter regions of interest, ensuring specificity for the subsequent analysis. The qRT-PCR was then conducted using these primers (Table S1) to amplify and quantify the enriched DNA fragments, allowing for the assessment of protein-DNA interactions within the chromatin.

### Nascent RNA capture

We used the clickit RNA Capture Kit (C10365, Invitrogen, Carlsbad, CA, USA) in EC cells to capture nascent RNA. The nascent RNA was labeled biotinylated with 5-ethyluridine (EU), and the labeled RNA was separated by magnetic streptavidin beads for subsequent RT-qPCR.

### RNA stability measurement

Actinomycin D (HY-17,559, MCE, Shanghai, China) was introduced into the culture medium of EC cells to impede transcriptional activity. Cells were collected at 0, 3, and 6 h after actinomycin D was added. The expression levels of specific RNAs at these time intervals were quantitatively assessed by RT-qPCR. The degradation rate of the RNA was deduced by comparing the expression levels over time. Compared with 0 h, the half-life was measured when the RNA expression reached 50%.

### Xenograft tumor experiment in nude mice

In this in vivo study, female four-week-old nude mice without thymus (BALB/ C) were purchased from Huafukang Biotechnology Co., Ltd., (Beijing, China). All procedures involving these animals were meticulously designed and conducted in strict adherence to the Animal Welfare Act, receiving full approval from the Ethics Committee of China Medical University (2020PS190K). To further confirm the results of WTAP promoting EC cisplatin sensitivity in vivo, we inoculated WTAP(+) or WTAP(+) NC ECSCs subcutaneously in nude mice. During subcutaneous implantation, 1 × 10^6^ stably transfected and expressed ECSCs cells were injected subcutaneously into the right axilla. Nude mice with subcutaneous tumors were treated with cisplatin (3 mg /kg per mouse) or saline once a week for 4 weeks (a total of 4 injections) when the tumors were first palpable for 1 week. The results further confirm the role of the WTAP/EGR1/PTEN axis on EC in vivo, nude mice were randomly divided into six groups: control group, WTAP (+) group, EGR1 (+) group, PTEN (+) group, WTAP (+) + EGR1 (+) group, and EGR1 (+) + PTEN (+) group. During subcutaneous implantation, 1 × 10^6^ stably transfected and expressed cells (Ishikawa and ECSCs) were injected subcutaneously into the right armpit. The tumor volume was evaluated every 4 days and observed continuously for 28 days. Tumor volume was calculated according to the following formula: tumor volumes (mm3) = length × width^2^/2.

### Statistical analysis

Data from the study are expressed as the mean ± standard deviation (SD) of three independent experiments. Statistical analyses were conducted using GraphPad Prism 8 software (Graphpad, San Diego, CA, USA). The distribution normality of the dataset was assessed via D’Agostino-Pearson and Shapiro-Wilk tests. A non-paired two-sided t-test was used for data with a normal distribution, and a nonparametric Mann Whitney test was used to compare the two groups. When assessing differences among more than two groups, one-way Analysis of Variance (ANOVA) was applied. *P* < 0.05 was statistically significant.

## Results

### WTAP and m6A modifications are reduced in EC and function as signature features of ECSCs

**Fig. 1:**
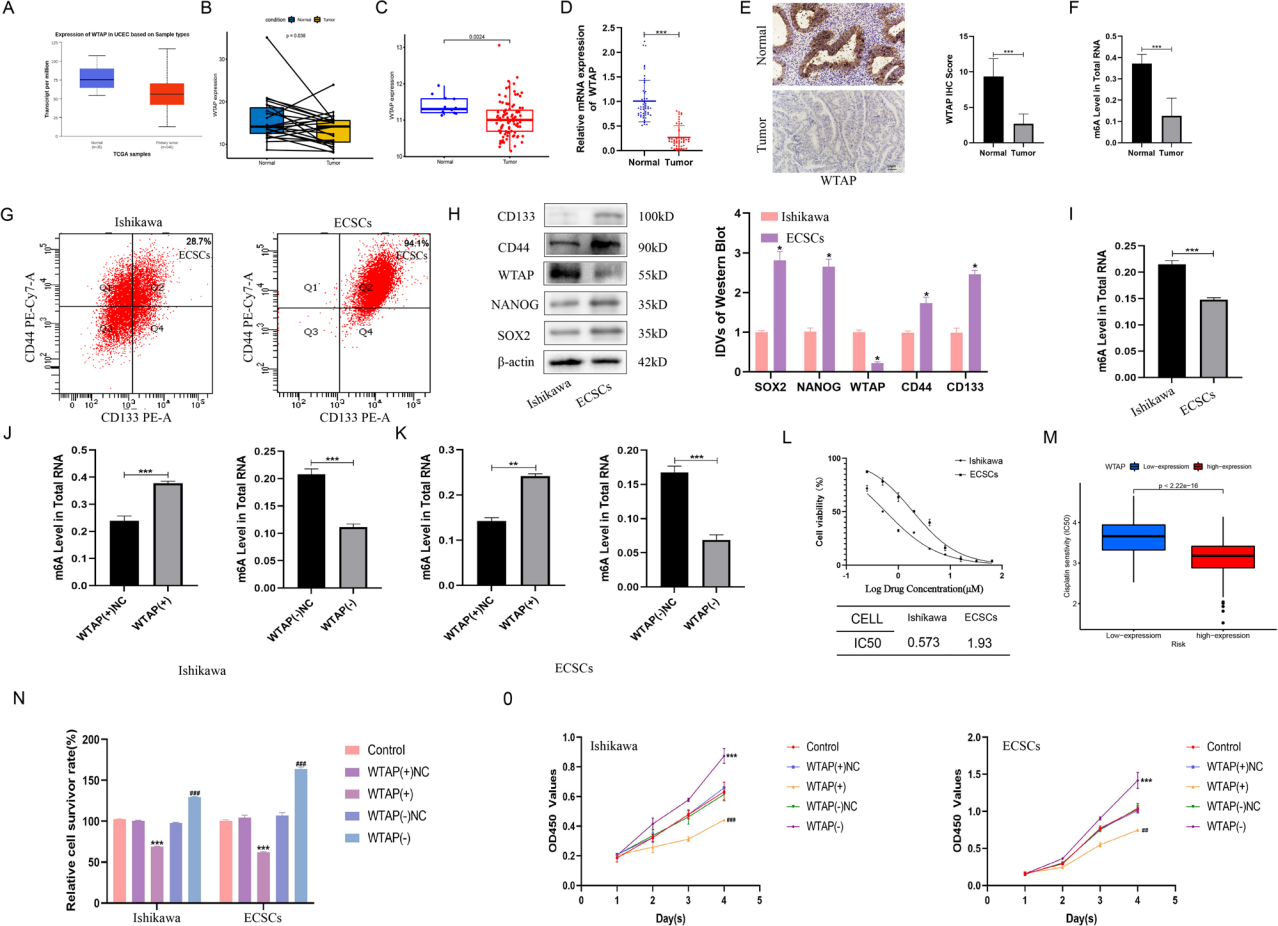
WTAP is down-regulated in endometrial cancer (EC) and promotes the malignant behavior of EC cells (ECCs) and ECSCs (ECSCs). (**A**)The relative expression of WTAP in uterine corpus EC (*n* = 546) and normal endometrial tissue (*n* = 35) based on data from the UALCAN (ualcan.path.uab.edu) database. ***P < 0.001 (**B**) The relative expression of WTAP in uterine corpus EC (*n* = 23) and paired normal endometrial tissue (*n* = 23) based on data from the TCGA database. (**C**) The relative expression of WTAP in EC (*n* = 91) and normal tissues (*n* = 12) based on data from the GEO database (GSE17025). (**D**) WTAP expression was evaluated in EC tissues (*n* = 50) and normal endometrial tissues (*n* = 50) by qRT-PCR. (**E**) WTAP expression levels in EC tissues (*n* = 50) compared to normal tissues (*n* = 50) detected using immunohistochemistry. Data are presented as the means ± SEM, ***P < 0.001. (**F**) The m6A levels in EC tissues (*n* = 50) compared to normal tissues (*n* = 50). Data are presented as the means ± SEM, ***P < 0.001. (**G**) The CD44+/CD133 + cells obtained through sorting were considered as ECSCs (**H**) The expression of CD44, CD133, WTAP, SOX2, and NANOG in Ishikawa cell lines and ECSCs detected using western blotting. (**I**) The m6A levels in Ishikawa cell lines and ECSCs (*n* = 3). Data are presented as the means ± SEM, ***P < 0.001. (**J**-**K**) m6A level in total RNA of Ishikawa cell lines and ECSCs (*n* = 3), WTAP overexpression, and WTAP knockdown. (**L**) Sensitivity of Ishikawa cell lines and ECSCs to cisplatin. (**M**) The “pRRophetic” in R was used to predict the correlation between WTAP expression and cisplatin resistance. (**N**) Effects of WTAP on cisplatin resistance assessed using the CCK8 assay. (**O**) Effect of WTAP on proliferation assessed using the CCK8 assay

In recent years, more and more studies have shown that m6A modification plays an important role in CSCs. First, we are interested in the function of m6A modification in the ECCs and ECSCs. The UALCAN platform (EC = 546, normal = 35), the TCGA of EC and its matching tissue(EC = 23, normal = 23), and GEO database (GSE17025; EC = 91, normal = 12) were used to analyze the expression of classical m6A related enzymes WTAP, METTL3, METTL14, FTO and ALKBH5.The results showed that all the data in the three databases supported the down-regulation of WTAP expression in EC, while METTL3, METTL14, FTO and ALKBH5 showed no difference or contradictory results in the three databases (Fig. S1). To verify the expression of WTAP in EC, The UALCAN platform (EC = 546, normal = 35) was used to visualize the amount of WTAP expression and the results showed that WTAP was down-regulated in EC (Fig. 1A). We retrieved data pertaining to UCEC from the TCGA database, and the analysis results revealed downregulated expression of WTAP in EC tissues (*n* = 23) compared to paired normal tissues (*n* = 23) (Fig. 1B). An additional dataset, GSE17025, was obtained from the GEO dataset, and the analysis results also showed that WTAP expression was downregulated in EC tissues(*n* = 91) compared to normal tissues (*n* = 12) (Fig. 1C). This trend of reduced WTAP expression in EC was further confirmed through qRT‒PCR analyses of our own samples (Fig. 1D). Decreased WTAP was associated with FIGO stage, lymph node metastasis, and LVSI progression (Table S4). Immunohistochemical evaluations also confirmed these findings, with WTAP protein levels being notably lower in EC tissues than in normal tissues (Fig. 1E). Given the established role of WTAP as a canonical m6A methylase that is instrumental in modulating m6A modification levels, we investigated alterations in RNA m6A methylation in EC samples. The analyses revealed a significant reduction in m6A methylation levels in EC tissues compared to normal tissues, a pattern that mirrors the observed WTAP downregulation (Fig. 1F). These results suggest that WTAP is low in EC and correlated with clinicopathological parameters, which may inhibit the occurrence and development of EC. In recent years, more and more studies have shown that m6A modification is related to CSCs [20], so we further explored whether WTAP is involved in stemness maintenance of ECSCs. Subsequent sorting of Ishikawa cells yielded a population enriched for CD44 + and CD133 + stem cells. The proportion of CD44 + and CD133 + stem cells in Ishikawa was 28.7%, and these cells were ECSCs. The results of our re-identification of these cells showed that 94.1% of the cells were CD44 + and CD133+, which confirmed the successful sorting of ECSCs (Fig. 1G). We used Western blotting to detect the downregulated expression of WTAP in endometrial cancer stem cells compared to that in Ishikawa cells, while other stem cell indicators, such as CD44, CD133, SOX2 and NANOG, were upregulated in ECSCs (Fig. 1H). An overall decrease decline in RNA m6A modification levels was found in ECSCs relative to Ishikawa cells (Fig. 1I). We further knocked down and overexpressed WTAP in Ishikawa cells and ECSCs and found that the m6A modification of total RNA also decreased and increased, respectively (Fig. 1J-K). These cumulative findings suggest a pivotal role of WTAP and m6A modification in the stemness of ECCs, with a marked decrease in both parameters within the ECSCs subset, potentially indicating that the regulatory capacity of WTAP exceeds that of m6A in fostering the stem-like qualities of EC cells.

### Downregulation of WTAP is required to maintain the EC stem cell (ECSC) phenotype

**Fig. 2:**
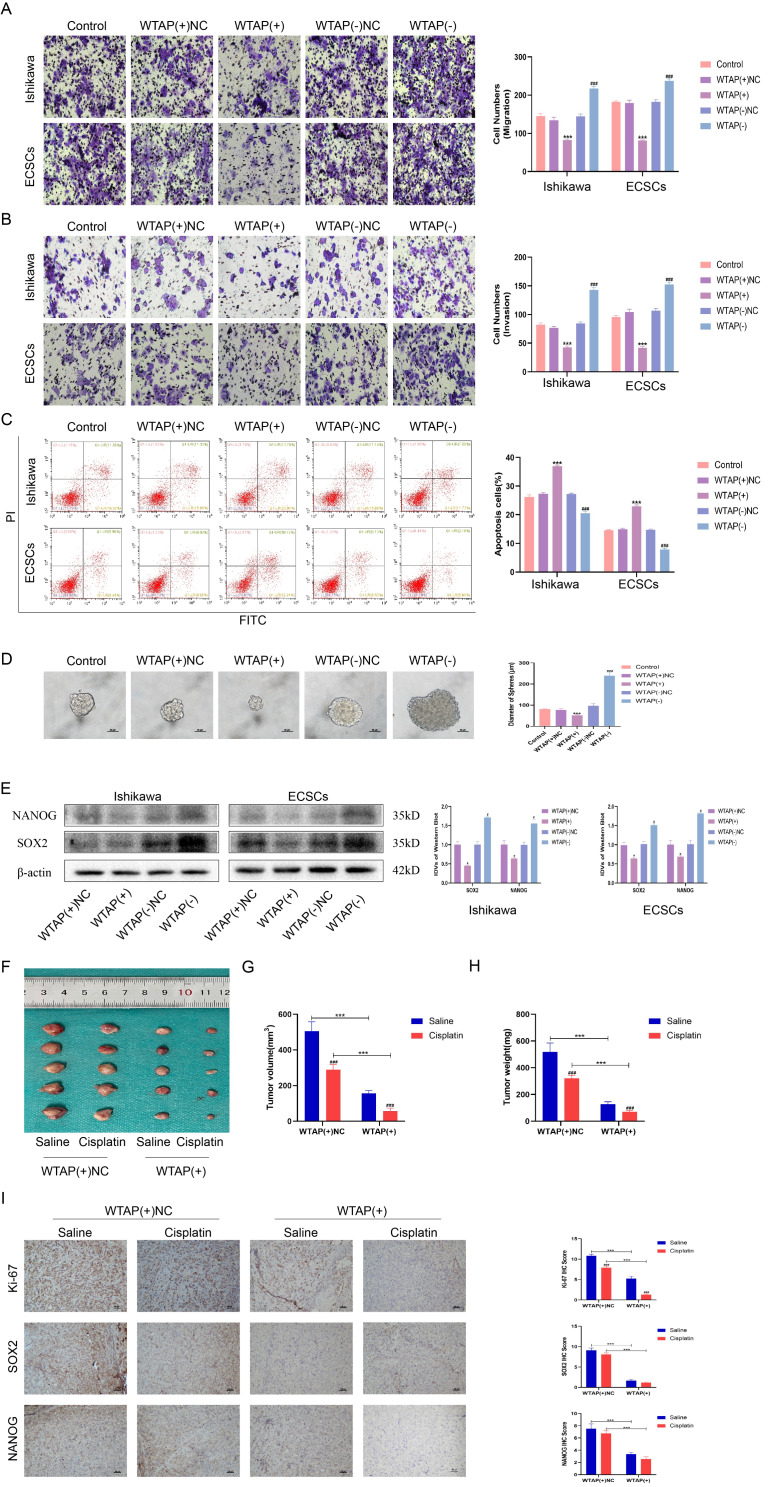
WTAP is down-regulated in EC and promotes the malignant behavior of ECCs and ECSCs. **(A)** Effects of WTAP on migration assessed using the Transwell assay (Scale bars, 100 μm). **(B)** Effects of WTAP on invasion assessed using the Transwell assay (Scale bars, 100 μm). **(C)** Effects of WTAP on apoptosis assessed using flow cytometry analysis. **(D)** Effects of WTAP on self-renewal capacity assessed using serial sphere formation assay (Scale bars, 50 μm). **(E)** Effects of WTAP overexpression or knockdown on SOX2 and NANOG expression assessed using western blotting (*n* = 3, each group). **(F)** The nude mice carrying tumors from the respective groups are shown. The sample tumors from the respective groups are shown (*n* = 3, each group). **(G)** Tumor volumes of 4 groups from F. **(H)** Tumor weight of 4 groups from E. **(I)** immunohistochemical staining on the xenograft tumor from nude mice was performed to detected the effects of Ki-67, SOX2 and NANOG expression. Data are presented as the means ± SD (*n* = 3, each group), *P < 0.05, **P < 0.01, ***P < 0.001 vs. WTAP(+) NC group; #*P* < 0.05, ##*P* < 0.01 and ###*P* < 0.001 vs. Saline group

Subsequent assessments of the IC50 values for cisplatin on delineated populations of ECSCs and Ishikawa cells showed that ECSCs exhibited increased resistance to cisplatin (Fig. 1L). Utilizing the “pRRophetic” package in R for predicting drug sensitivity in relation to WTAP expression levels revealed that samples characterized by diminished WTAP expression exhibited heightened resistance to cisplatin (Fig. 1M). CCK-8 assays confirmed that downregulation of WTAP fortified cisplatin resistance in both ECCs and ECSCs, whereas WTAP overexpression attenuated this resistance (Fig. 1N). Furthermore, CCK8 assays elucidated that WTAP overexpression curtailed the proliferative capacities of ECCs and ECSCs, whereas WTAP knockdown had the opposite effect, enhancing their proliferation. (Fig. 1O, Fig. S2A). Transwell assays were used to assess the invasion and migration of ECCs and ECSCs. The results showed that overexpression of WTAP inhibited the invasion and migration of ECCs and ECSCs, while knockdown of WTAP promoted the invasion and migration of ECCs and ECSCs (Fig. 2A-B, Fig. S2B-C). Apoptosis analyses revealed that WTAP overexpression promoted apoptosis, whereas its knockdown inhibited apoptosis in both ECCs and ECSCs (Fig. 2C). Sphere formation assays, which assess the self-renewal potential, indicated that WTAP reduction was integral to the maintenance of ECSCs characteristics, with WTAP overexpression inhibiting and its knockdown promoting stem cell self-renewal (Fig. 2D). Western blot analyses targeting the stemness markers NANOG and SOX2 after WTAP modulation demonstrated that WTAP knockdown amplified, while WTAP overexpression reduced, the expression of these stemness indicators, suggesting an increase or decrease in stem cell attributes (Fig. 2E). In addition, WTAP was knocked down in Ishikawa and ECSCs at the same time, and WB analysis results showed that SOX2 and NANOG were elevated in the WTAP knockout group (Fig. S1D). In vivo, nude mice harboring EC xenografts were administered cisplatin (3 mg /kg) or saline weekly over a span of four weeks, resulting in a notable reduction in the tumor volume in the cisplatin-treated cohort compared to that in the saline-treated cohort (Fig. 2F-H). Moreover, WTAP overexpression in xenografts contributed to reduced tumor growth in both the cisplatin and saline groups compared with that in the WTAP(+) NC counterparts (Fig. 2F-H). Immunohistochemical results showed that the expression levels of Ki-67, NANOG and SOX2 in the cisplatin group were decreased compared with those in the normal saline group (Fig. 2I). Immunohistochemical analyses of the xenograft tissues revealed a decrease in Ki-67, NANOG, and SOX2 expression in the cisplatin group relative to that in the saline group and a decrease in Ki-67, NANOG, and SOX2 expression in the WTAP(+) group compared to that in the WTAP(+) NC group across both treatment regimens (Fig. 2I). These in vivo findings underscore the efficacy of cisplatin in inhibiting xenograft tumor growth, with WTAP overexpression not only hindering tumor proliferation but also amplifying tumor sensitivity to cisplatin.

### WTAP affects the stability of EGR1 mRNA by regulating m6A modifications on its mRNA

**Fig. 3:**
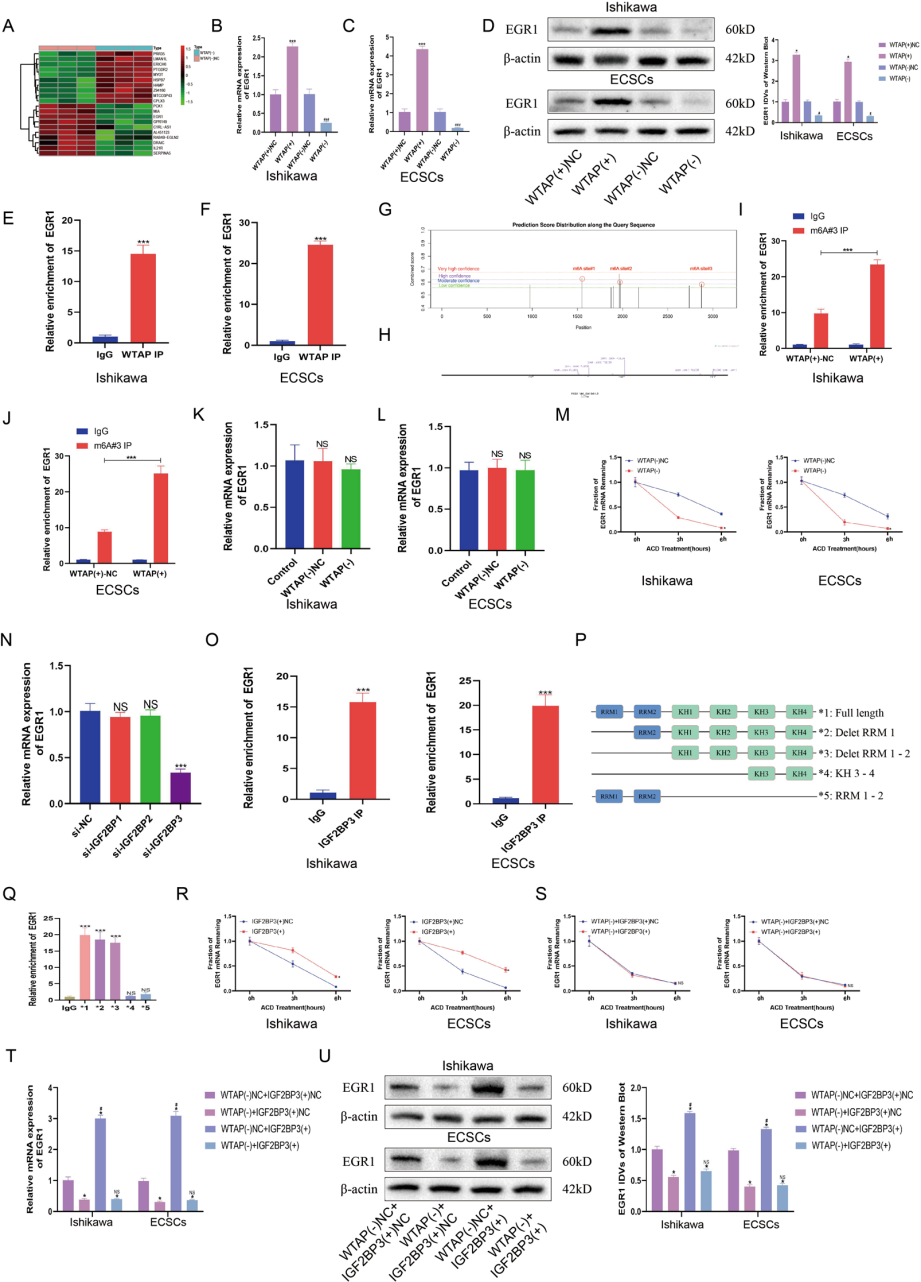
WTAP-mediated m6A modification of EGR1 promotes its stability. **(A)** RNA sequencing detected transcriptomic changes in ECSCs during WTAP knockdown (*n* = 3 per group, Top 20 genes). **(B)** Effects of WTAP overexpression or knockdown on mRNA of EGR1 expression assessed using PCR in Ishikawa cell lines. **(C)** Effects of WTAP overexpression or knockdown on mRNA of EGR1 expression assessed using PCR in ECSCs. **(D)** Effects of WTAP overexpression or knockdown on EGR1 expression assessed using western blotting in Ishikawa cell lines and ECSCs. **(E-F)** An enrichment of EGR1 mRNA in WTAP immunoprecipitated samples via RNA immunoprecipitation (RIP) assay (*n* = 3) in Ishikawa cell lines and ECSCs. **(G)** The enriched and specific m6A peak distribution of EGR1 transcripts predicted by SRAMP. Red circles indicate enrichment peaks in m6A. **(H)** Specific primers were designed according to the m6A enrichment site on the EGR1 transcript. **(I-G)** m6A enrichment in EGR1 transcripts in WTAP(+) NC and WTAP(+) cells using MeRIP-qPCR in Ishikawa cell lines and ECSCs. **(K-L)** Expression of nascent EGR1 was measured via RT-qPCR after WTAP knockdown (*n* = 3, each group). **(M)** Half-life of EGR1 was measured by RT-qPCR after actinomycin D treated in Ishikawa cell lines and ECSCs (*n* = 3, each group). **(N)** Effects of IGF2BP1/2/3 knockdown on mRNA of EGR1 expression assessed using PCR in Ishikawa cell lines. **(O)** An enrichment of EGR1 mRNA in IGF2BP3 immunoprecipitated samples RIP assay (*n* = 3, each group) in Ishikawa cell lines and ECSCs. **(P-Q)** Deletion mapping for the domains of IGF2BP3 that bind to mRNA of EGR1, RIP analysis for EGR1 enrichment in cells transiently transfected with plasmids containing the indicated FLAG-tagged full-length or truncated constructs. **(R-S)** Half-life of EGR1 was measured by RT-qPCR after actinomycin D treated in Ishikawa cell lines and ECSCs (*n* = 3, each group). **(T)** The effects of WTAP and IGF3BP3 co-transfer on EGR1 mRNA expression in Ishikawa cell lines and ECSCs were detected by PCR (*n* = 3). **(U)** The effects of WTAP and IGF3BP3 co-transfer on EGR1expression in Ishikawa cell lines and ECSCs were detected by western blotting (*n* = 3, each group)

To elucidate how WTAP depletion promotes stemness in ECSCs and augments the proliferation and migration of EC cells, we performed transcriptomic sequencing of the WTAP(-) and WTAP(-) NC groups. The sequencing data revealed the top 10 upregulated and downregulated genes in the WTAP(-) cohort relative to the WTAP(-) NC group (Fig. 3A). We predicted these genes on the RM2TARGET website (http://rm2target.canceromics.org/) to predict WTAP binding to these genes and m6A modification and finally obtained the optimal gene, EGR1 (the results showed that WTAP and EGR1 mRNA could bind, and the confidence score was the highest). There were abundant m6A modifications on EGR1 mRNA, and WTAP was correlated with EGR1 in UCEC (Table S5). Reductions in EGR1 mRNA were notably observed following WTAP knockdown in both ECCs and ECSCs, while EGR1 mRNA levels were elevated in conjunction with WTAP overexpression (Fig. 3B-C). These alterations in WTAP levels correspondingly influenced EGR1 mRNA and protein expression, with WTAP overexpression leading to an increase in EGR1 protein levels, and its knockdown resulting in a decrease (Fig. 3D). RIP experiments confirmed the capacity of WTAP to bind to EGR1 mRNA in both ECCs and ECSCs (Fig. 3E-F). We further used the SRAMP database (https://www.cuilab.cn/sramp) to predict the m6A modification site on the transcript of EGR1 (Fig. 3G). Then, we designed MeRIP-specific primers according to the m6A enrichment site of the EGR1 transcripts (Fig. 3H). MeRIP assays demonstrated that WTAP modulates m6A modifications on EGR1 transcripts in both the ECCs and ECSCs populations (Fig. 3I-J). Further investigations aimed at delineating the role of WTAP in the m6A modification of EGR1 involved the use of a nascent RNA capture technique, which revealed that WTAP knockdown did not significantly impact nascent EGR1 mRNA levels in either ECCs or ECSCs (Fig. 3K-L). However, RNA stability assays indicated a marked decrease in EGR1 mRNA stability in the WTAP(-) group compared to the WTAP(-) NC group in both the ECCs and ECSCs, highlighting the pivotal role of WTAP in regulating EGR1 mRNA stability through m6A modifications, thereby influencing EC cell stemness and oncogenic properties (Fig. 3M).

### IGF2BP3 acts as an m6A reader and collaborates with WTAP to regulate EGR1

The m6A modification of EGR1 mRNA by WTAP was found to significantly influence its mRNA stability. Within the m6A reading framework, the IGF2BP family is a classic set of readers, suggesting that IGF2BPs may act as readers for EGR1 mRNA. Targeted knockdown experiments of IGF2BP1, IGF2BP2, and IGF2BP3 were conducted to determine their influence on EGR1 mRNA levels. The results indicated that while the suppression of IGF2BP1 and IGF2BP2 did not affect EGR1 mRNA levels, the knockdown of IGF2BP3 led to a notable reduction in EGR1 mRNA content (Fig. 3N). We further used RIP-qPCR to show that IGF2BP3 can bind to EGR1 mRNA (Fig. 3O). The structural composition of IGF2BP3, which features two RNA recognition motifs (RRMs) and four K homology domains (KHs) [36], prompted the generation of various full-length and fragmented IGF2BP3 constructs with Flag tags, which were subsequently introduced into the HEK293T cell line (Fig. 3P). Subsequent RIP-qPCR assays highlighted the predominant enrichment of EGR1 mRNA in the KH1-2 domain of IGF2BP3 (Fig. 3Q). RNA stability assessments revealed that EGR1 mRNA stability was significantly greater in the IGF2BP3(+) cohort than in the IGF2BP3(+) NC cohort among ECCs and ECSCs (Fig. 3R). To explore the synergistic regulation of EGR1 mRNA by WTAP and IGF2BP3, we established experimental groups with combined WTAP knockdown and IGF2BP3 overexpression. Stability assay showed no discernible difference in EGR1 mRNA stability between the WTAP(-) + IGF2BP3(+) and WTAP(-) + IGF2BP3(+) NC groups (Fig. 3S). Finally, qRT‒PCR was used to verify the changes in EGR1 mRNA levels. Compared with that in the WTAP(-) NC + IGF2BP(+) NC group, EGR1 mRNA in the WTAP(-) + IGF2BP3(+) NC group was decreased. The EGR1 mRNA level was increased in the WTAP(-) NC + IGF2BP3(+) group. However, there was no change in the EGR1 mRNA content in the WTAP(-) + IGF2BP3(+) group compared with that in the WTAP(-) + IGF2BP3(+) NC group, and the EGR1 protein levels were also consistent with the RNA levels (Fig. 3T-U). These findings revealed that IGF2BP3, as an m6A reader, can recognize m6A-modified EGR1 mRNA orchestrated by WTAP, thereby altering its RNA stability. However, the decrease in WTAP, even with concurrent IGF2BP3 overexpression and the resulting reduction in m6A modifications on EGR1 mRNA, did not affect EGR1 mRNA stability. This finding highlights the dependency of the binding affinity of IGF2BP3 for EGR1 mRNA on the presence of m6A modifications facilitated by WTAP, revealing the intricate regulatory mechanisms governing mRNA stability in the context of ECSCs maintenance and oncogenic progression.

### Downregulation of EGR1 is required to maintain the ECSC phenotype

**Fig. 4:**
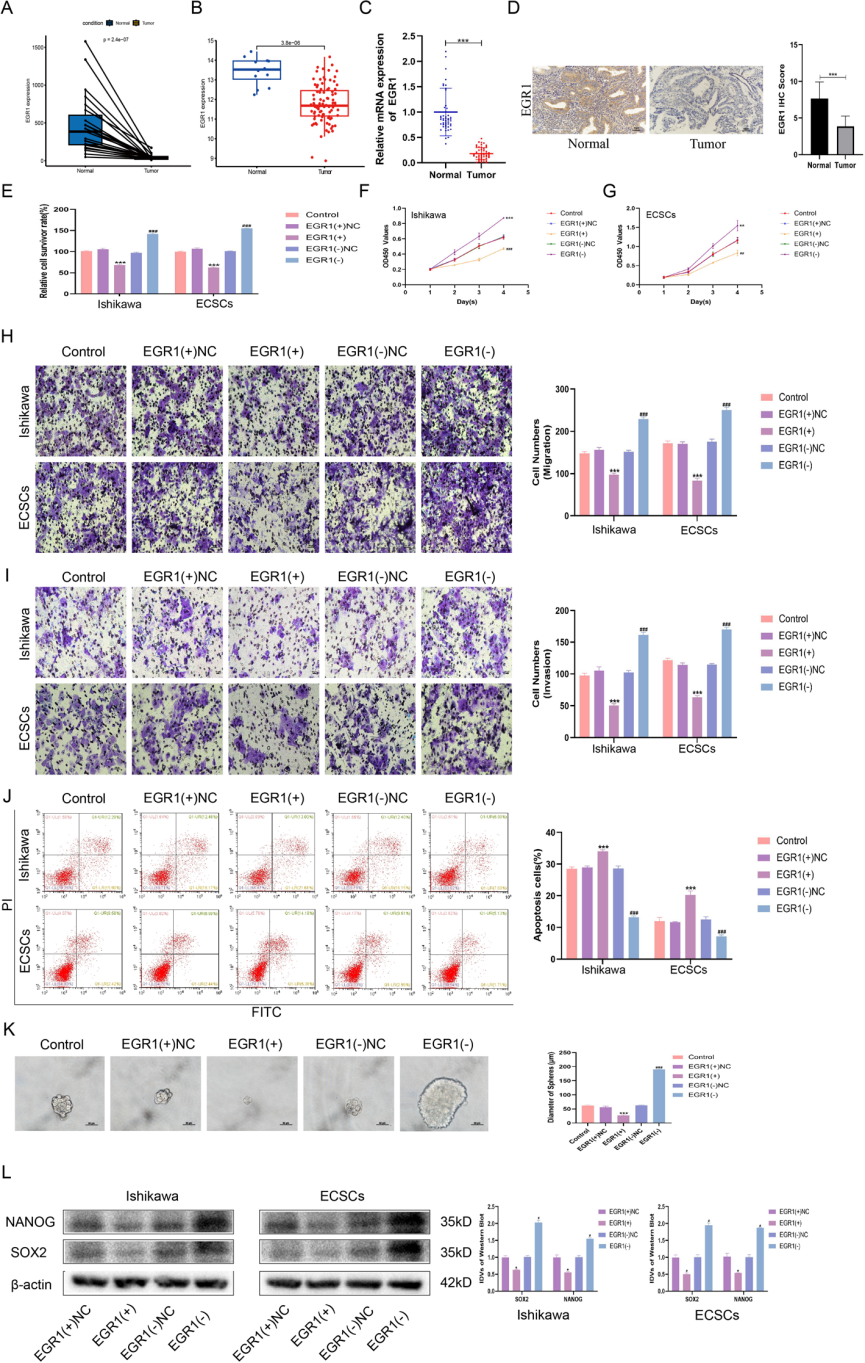
EGR1 is down-regulated in EC and promotes the malignant behavior of ECCs and ECSCs. **(A)** The relative expression of EGR1 in EC (*n* = 23) and paired normal endometrial tissue (*n* = 23) based on data from the TCGA database. **(B)** The relative expression of EGR1 in EC (*n* = 91) and normal tissues (*n* = 12) based on data from the GEO database (GSE17025). **(C)** EGR1 expression was evaluated in EC tissues (*n* = 50) and normal endometrial tissues (*n* = 50) by qRT-PCR. **(D)** EGR1 expression levels in EC tissues (*n* = 50) compared to normal tissues (*n* = 50) detected using immunohistochemistry. Data are presented as the means ± SEM, ***P < 0.001. **(E)** Effects of EGR1 on cisplatin resistance assessed using the CCK8 assay. **(F-G)** Effect of EGR1 on proliferation assessed using the CCK8 assay. **(H)** Effects of EGR1on migration assessed using the Transwell assay (Scale bars, 100 μm). **(I)** Effects of EGR1 on invasion assessed using the Transwell assay (Scale bars, 100 μm). **(G)** Effects of EGR1 on apoptosis assessed using flow cytometry analysis. **(K)** Effects of EGR1 on self-renewal capacity assessed using serial sphere formation assay (Scale bars, 50 μm). **(L)** Effects of EGR1 overexpression or knockdown on SOX2 and NANOG expression assessed using western blotting (*n* = 3, each group). Data are presented as the means ± SD (*n* = 3, each group), *P < 0.05, **P < 0.01, ***P < 0.001 vs. EGR1(+)NC group; #*P* < 0.05, ##*P* < 0.01 and ###*P* < 0.001 vs. EGR1(-)NC group

Analysis of data from TCGA-UCEC paired samples revealed a significant reduction in EGR1 expression in EC relative to normal tissue (Fig. 4A). This finding was corroborated by data from the GSE17025 dataset, which also demonstrated decreased EGR1 expression in EC (Fig. 4B). The results of the confirmatory qRT‒PCR analysis of our own samples aligned with these observations, showing lower EGR1 levels in EC tissues than in normal tissues (Fig. 4C). Decreased EGR1 was associated with FIGO stage, lymph node metastasis, and lymphatic metastatic progression (Table S6). Immunohistochemical evaluations further substantiated the reduced expression of EGR1 in EC tissue relative to normal tissue (Fig. 4D). The downregulation of EGR1 was identified as necessary for the maintenance of the ECSC phenotype. Functional assays indicated that EGR1 overexpression in ECCs and ECSCs increased cellular sensitivity to cisplatin, whereas EGR1 knockdown reduced this sensitivity (Fig. 4E). CCK-8 assays revealed that EGR1 overexpression decreased cell proliferation, whereas EGR1 knockdown increased proliferation in both ECCs and ECSCs (Fig. 4F-G, Fig. S2D). Transwell assays assessing the cell invasive and metastatic capabilities showed that EGR1 overexpression hindered these processes, while EGR1 knockdown promoted them in ECCs and ECSCs (Fig. 4H-I, Fig. S2E-F). Apoptotic analyses demonstrated that EGR1 overexpression augmented apoptosis, whereas EGR1 knockdown impeded apoptotic events in both ECCs and ECSCs (Fig. 4J). Sphere formation assays, employed to evaluate the self-renewal capacity of ECSCs, indicated that reduced EGR1 expression is characteristic of ECSC maintenance. Overexpression of EGR1 inhibited, and knockdown of EGR1 promoted, the self-renewal of ECSCs (Fig. 4K). Western blot assays further validated these findings, showing that EGR1 overexpression suppressed, while EGR1 knockdown increased, the expression of the stemness markers NANOG and SOX2. These comprehensive analyses elucidated the critical role of EGR1 in regulating various cellular processes in EC, including sensitivity to chemotherapy, proliferation, invasion, metastasis, apoptosis, and the maintenance of stem cell properties (Fig. 4L).

### Downregulation of WTAP destabilizes EGR1 and promotes the malignant biological behavior of ECCs and ECSCs

**Fig. 5:**
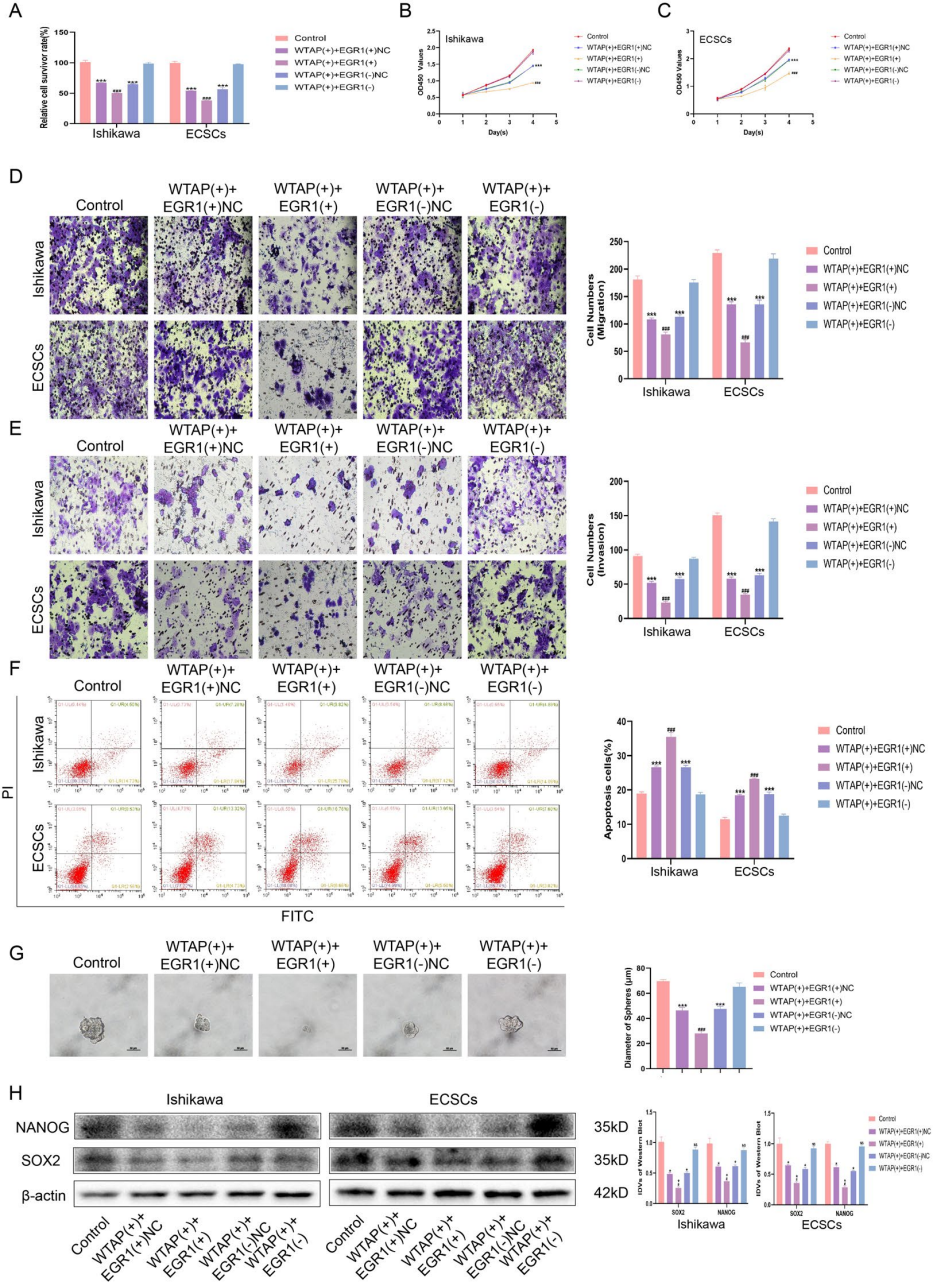
WTAP regulates the biological behavior of ECCs and ECSCs by stabilizing mRNA of EGR1. **(A)** Effects of WTAP and EGR1 on cisplatin resistance assessed using the CCK8 assay. **(B-C)** Effect of WTAP and EGR1 on proliferation assessed using the CCK8 assay. **(D)** Effects of WTAP and EGR1on migration assessed using the Transwell assay (Scale bars, 100 μm). **(E)** Effects of WTAP and EGR1 on invasion assessed using the Transwell assay (Scale bars, 100 μm). **(F)** Effects of WTAP and EGR1 on apoptosis assessed using flow cytometry analysis. **(G)** Effects of WTAP and EGR1 on self-renewal capacity assessed using serial sphere formation assay (Scale bars, 50 μm). **(H)** Effects of WTAP and EGR1 on SOX2 and NANOG expression assessed using western blotting (*n* = 3, each group). Data are presented as the means ± SD (*n* = 3, each group), *P < 0.05, **P < 0.01, ***P < 0.001 vs. Control group; #*P* < 0.05, ##*P* < 0.01 and ###*P* < 0.001 vs. WTAP(+) + EGR1(+) NC group

These findings revealed that the decrease in WTAP and EGR1 synergistically exacerbates the malignant phenotype of both ECCs and ECSCs. The findings elucidate that the diminution of WTAP and EGR1 synergistically exacerbates the malignant phenotype of both ECCs and ECSCs. In terms of cellular resistance to cisplatin, cellular proliferation, invasion, and metastasis, WTAP overexpression alone exhibited inhibitory effects. The concurrent overexpression of WTAP and EGR1 intensified this inhibition. However, the suppression of EGR1 expression reversed the inhibitory impact of WTAP overexpression on resistance to cisplatin, cell proliferation, invasion, and metastasis (Fig. 5A-E, Fig. S2G-I). Apoptosis assays revealed that WTAP overexpression independently promoted apoptotic cell death, an effect that was further potentiated by the simultaneous overexpression of WTAP and EGR1. The reduction in EGR1 levels, on the other hand, mitigated the proapoptotic effect of WTAP overexpression (Fig. 5F). Sphere formation assays, which are indicative of stem cell self-renewal capacity, demonstrated that WTAP overexpression alone inhibited this ability. The combined overexpression of WTAP and EGR1 led to an even more pronounced inhibition of ECSCs self-renewal. Conversely, EGR1 knockdown alleviated the suppression of ECSCs self-renewal induced by WTAP overexpression (Fig. 5G). Western blot analyses corroborated the results of these functional assays, showing a decrease in the expression levels of the stemness markers NANOG and SOX2 in the WTAP overexpressing cohort. This decrease was further accentuated by the co-overexpression of WTAP and EGR1. However, the knockdown of EGR1 counterbalanced the reduction in NANOG and SOX2 levels caused by WTAP overexpression (Fig. 5H, Fig. S2J). Collectively, these results highlight the complex interplay between WTAP and EGR1 in modulating the malignant behaviors and stem cell properties of ECCs, suggesting potential therapeutic targets for inhibiting EC progression and increasing chemosensitivity.

### Downregulation of PTEN is required to maintain the ECSC phenotype

**Fig. 6:**
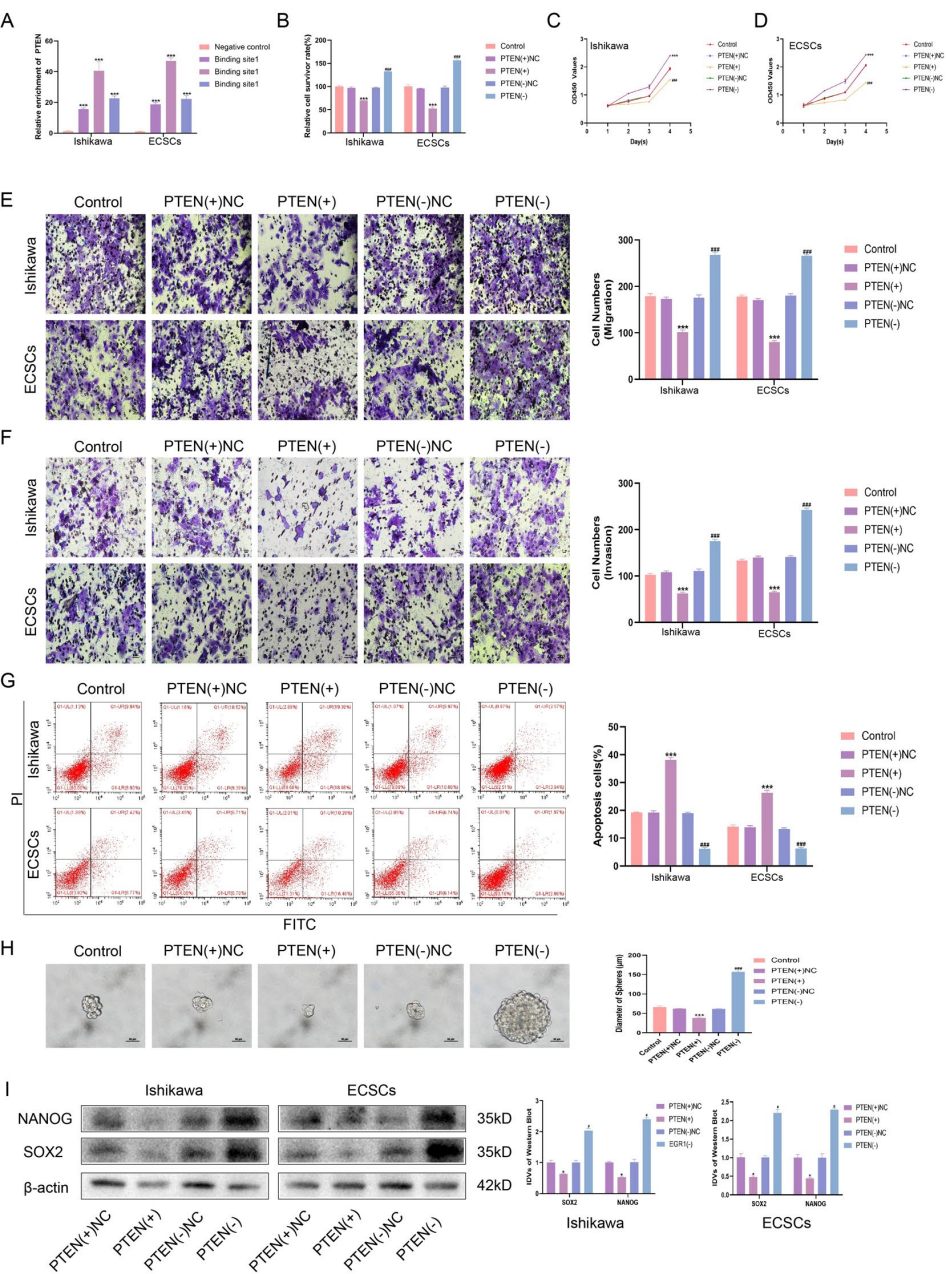
PTEN is down-regulated in endometrial cancer and promotes the malignant behavior of ECCs and ECSCs. **(A)** The binding of EGR1 and PTEN was detected by ChIP. **(B)** Effects of PTEN on cisplatin resistance assessed using the CCK8 assay. **(C-D)** Effect of PTEN on proliferation assessed using the CCK8 assay. **(E)** Effects of PTEN on migration assessed using the Transwell assay (Scale bars, 100 μm). **(F)** Effects of PTEN on invasion assessed using the Transwell assay (Scale bars, 100 μm). **(G)** Effects of PTEN on apoptosis assessed using flow cytometry analysis. **(H)** Effects of PTEN on self-renewal capacity assessed using serial sphere formation assay (Scale bars, 50 μm). **(I)** Effects of PTEN on SOX2 and NANOG expression assessed using western blotting (*n* = 3, each group). Data are presented as the means ± SD (*n* = 3, each group), *P < 0.05, **P < 0.01, ***P < 0.0001 vs. PTEN(+)NC group; #*P* < 0.05, ##*P* < 0.01 and ###*P* < 0.001 vs. PTEN(-)NC group

Through a literature review, it was found that the function of EGR1 as a tumor suppressor gene mostly depends on the transcription of PTEN as a transcription factor to exert its tumor suppressor function, and the JASPAR database also showed that PTEN was the target of EGR1 [37, 38]. ChIP experiments were conducted to validate the capacity of EGR1 transcriptionally activate the quintessential tumor suppressor gene PTEN (Fig. 6A). PTEN, recognized universally as a critical tumor suppressor, frequently exhibits deletion or diminished expression in a substantial fraction of EC cases [39]. Overexpression of PTEN promoted the sensitivity of cells to cisplatin, while knockdown of PTEN decreased the sensitivity of ECCs and ECSCs to cisplatin (Fig. 6B). Overexpression of PTEN inhibited cell proliferation, invasion and metastasis, while knockdown of PTEN promoted cell proliferation, invasion and metastasis in ECCs and ECSCs (Fig. 6C-F, Fig. S3A-C). Overexpression of PTEN promoted apoptosis, and knockdown of PTEN inhibited apoptosis in ECCs and ECSCs (Fig. 6G). Overexpression of PTEN inhibited the self-renewal ability of ECSCs, while knockdown of PTEN promoted the self-renewal of ECSCs (Fig. 6H). Western blot analyses corroborated these findings, revealing a decrease in the expression of the stemness markers NANOG and SOX2 upon PTEN overexpression. In contrast, PTEN knockdown led to the upregulation of NANOG and SOX2 expression, highlighting the regulatory influence of PTEN on stem cell characteristics in EC (Fig. 6I).

### Downregulation of EGR1 reduces the amount of PTEN and promotes malignant biological behavior of ECCs and ECSCs

**Fig. 7:**
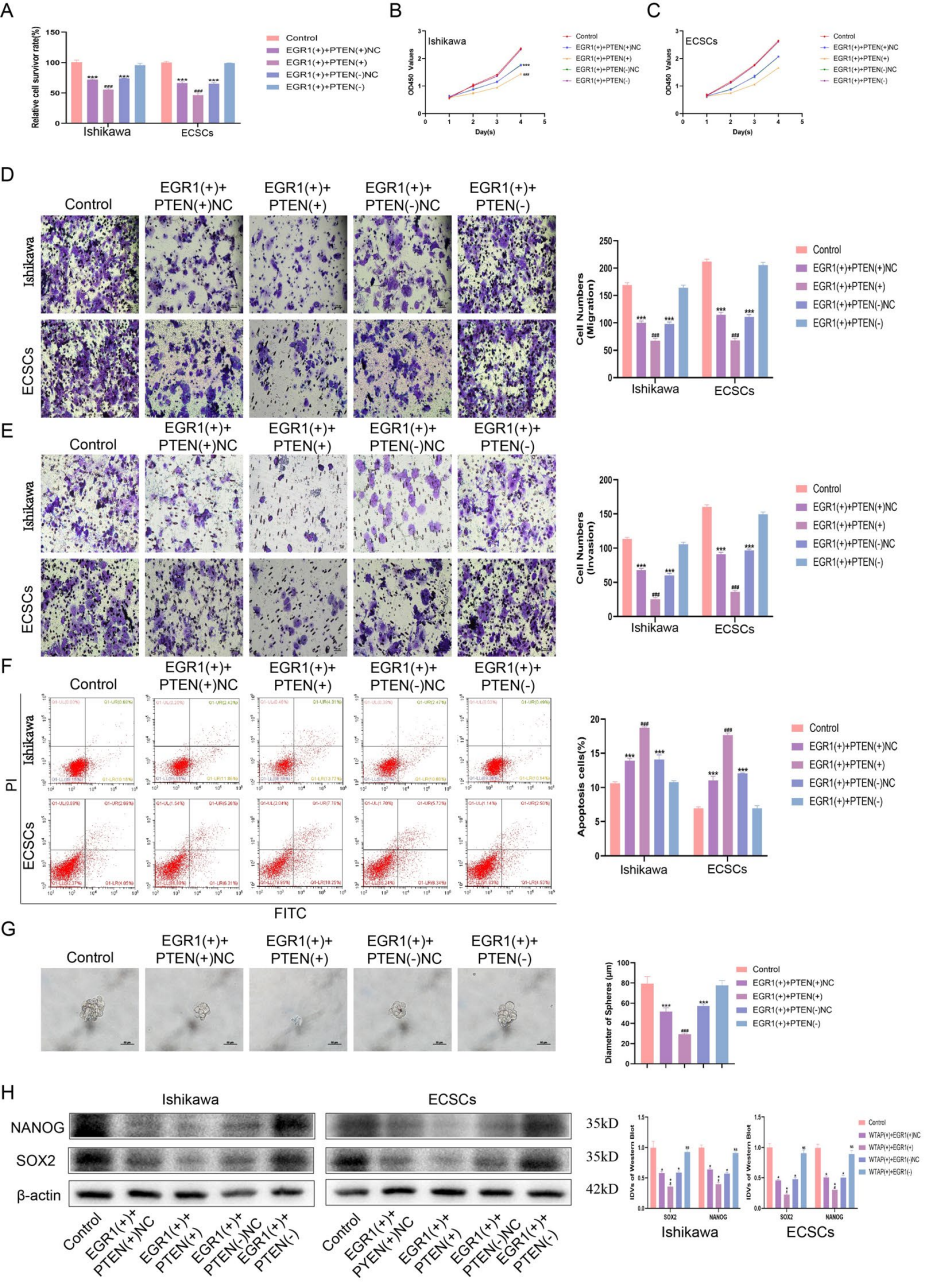
EGR1 regulates the biological behavior of ECSCs by transcribing PTEN. **(A)** Effects of EGR1 and PTEN on cisplatin resistance assessed using the CCK8 assay. **(B-C)** Effect of EGR1 and PTEN on proliferation assessed using the CCK8 assay. **(D)** Effects of EGR1 and PTEN on migration assessed using the Transwell assay (Scale bars, 100 μm). **(E)** Effects of EGR1 and PTEN on invasion assessed using the Transwell assay (Scale bars, 100 μm). **(F)** Effects of EGR1 and PTEN on apoptosis assessed using flow cytometry analysis. **(G)** Effects of EGR1 and PTEN on self-renewal capacity assessed using serial sphere formation assay (Scale bars, 50 μm). **(H)** Effects of EGR1 and PTEN on SOX2 and NANOG expression assessed using western blotting (*n* = 3, each group). Data are presented as the means ± SD (*n* = 3, each group), *P < 0.05, **P < 0.01, ***P < 0.001 vs. Control group; #*P* < 0.05, ##*P* < 0.01 and ###*P* < 0.001 vs. EGR1(+) + PTEN(+)NC group

To corroborate the role of the EGR1/PTEN axis in EC, we conducted extensive experiments. The results illustrated that, relative to that in the control group, EGR1 overexpression significantly reduced cellular resistance to cisplatin, as well as the proliferative, invasive, and migratory capacities of the cells. This inhibitory effect was further amplified when both EGR1 and PTEN were overexpressed, suggesting a synergistic interaction in suppressing malignant cellular behaviors. Conversely, PTEN knockdown mitigated the alterations induced by EGR1 overexpression, indicating that PTEN plays a crucial role in mediating the effects of EGR1 (Fig. 7A-E, Fig. S3D-F). Compared with that in the control group, EGR1 overexpression promoted apoptosis, while EGR1 and PTEN overexpression further increased apoptosis. Knocking down PTEN reversed the changes caused by EGR1 overexpression (Fig. 7F). Compared with those of the control group, the overexpression of EGR1 inhibited the self-renewal of ECSCs and inhibited the expression of NANOG and SOX2; the overexpression of RGR1 and PTEN further inhibited the self-renewal of ECSCs and the expression of NANOG and SOX2, while the knockout of PTEN reversed the changes caused by the overexpression of EGR1 (Fig. 7G-H, Fig. S3G). These findings collectively affirm the critical role of the EGR1/PTEN axis in modulating the oncogenic traits and stem cell-like properties of EC cells, revealing a compelling target for therapeutic intervention in endometrial carcinoma.

### Tumor xenografts in nude mice

**Fig. 8:**
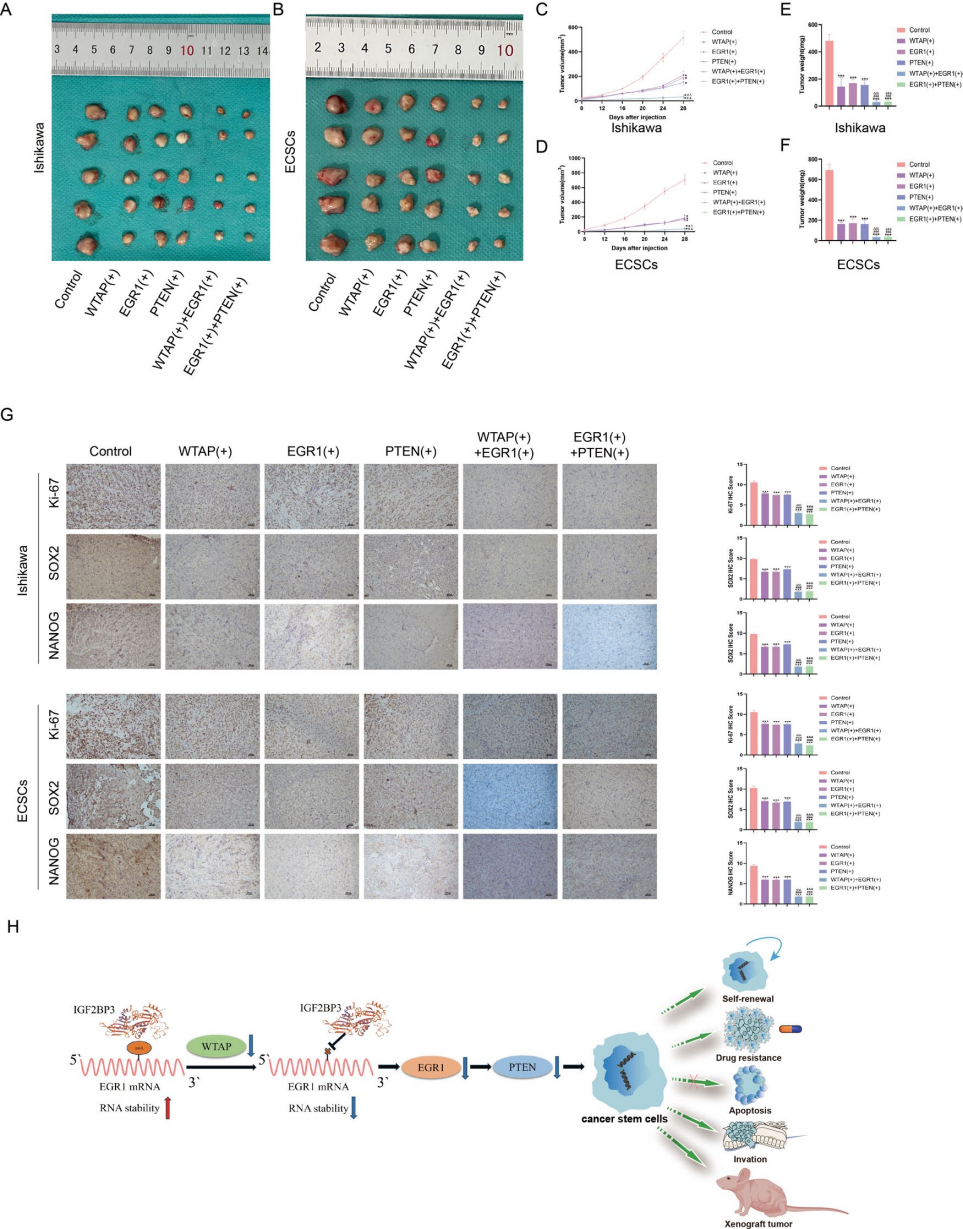
Simultaneous overexpression of WTAP, EGR1 and PTEN can inhibit tumor growth in vivo. **(A-B)** Subcutaneously xenografted nude mice injected with different treated cells are shown (*n* = 5, each group). **(C-D)** Tumor growth curves are shown. **(E-F)** Tumor average weight are shown; Tumor size was recorded every 4 days, and tumors were extracted at 28 days after injection. **(G)** immunohistochemical staining on the xenograft tumor from nude mice was performed to detected the effects of Ki-67, SOX2 and NANOG expression by overexpression of WTAP, EGR1 and PTEN; **(H)** mechanism diagram of WTAP / EGR1 / PTEN axis as a potential glycolytic metabolic regulator in EC. Data are presented as the means ± SEM (*n* = 3, each group), *P < 0.05, **P < 0.01, ***P < 0.001 vs. Control group, ###*P* < 0.001 vs.EGR1(+) group, △△△*P* < 0.01 vs. WTAP(+) group, &&&*P* < 0.001 vs. PTEN(+) group

To confirm the role of WTAP, EGR1, and PTEN in tumor growth in vivo, we constructed xenograft tumor models in nude mice by subcutaneous injection of overexpression of WTAP, EGR1, and PTEN in ECCs and ECSCs, respectively, or combined overexpression of WTAP + EGR1 or EGR1 + PTEN. The results showed that, compared with the control group, the group with overexpression of WTAP, EGR1 or PTEN alone had a significantly reduced the mean xenograft volume, while the group with combined overexpression of WTAP + EGR1 and EGR1 + PTEN had the smallest xenograft volume (Fig. 8A-D). Similarly, overexpression of WTAP, EGR1 or PTEN alone significantly reduced the mean weight of xenografts in nude mice compared with that of the control group, and the weight of the WTAP + EGR1 and EGR1 + PTEN combined overexpression group was the lowest (Fig. 8E-F). In addition, immunohistochemical staining of transplanted tumors in nude mice revealed that overexpression of WTAP, EGR1 or PTEN alone significantly decreased the expression of Ki-67, NANOG and SOX2 in transplanted tumors in nude mice compared with those in the control group, and the expression of Ki-67, NANOG and SOX2 was lowest in the WTAP + EGR1 and EGR1 + PTEN groups (Fig. 8G). Finally, we summarize the role of the WTAP/EGR1/PTEN axis in EC as a mechanistic map for regulating EC stem cell stem- maintenance (Fig. 8H). These experimental findings provide compelling evidence of the critical roles of WTAP, EGR1, and PTEN in regulating tumor growth and maintaining stem cell-like characteristics in EC. The results from the xenograft models affirm the potential of targeting the WTAP/EGR1/PTEN axis as a strategic approach for inhibiting tumor progression and stem cell-driven tumor propagation in EC.

## Discussion

The incidence of EC has steadily increased, positioning it as the most common gynecological malignancy in developed regions and certain urbanized locales within our country [40]. This increasing trend can be attributed to the increasing incidence of chronic conditions such as obesity, diabetes, and hypertension, which contribute to an annual increase of 1.9% in the number of patients with EC [41, 42]. Fortunately, many EC patients exhibit early symptoms such as abnormal vaginal bleeding, allowing early diagnosis and effective treatment. However, in cases where early symptoms of EC are subtle or overlooked and the disease progresses to the advanced stages of metastasis and invasion, the 5-year survival rate decreases to 16–45% [43]. Therefore, it is crucial to find new and effective therapeutic targets for treating EC.

In our study, both bioinformatics analyses and validation using independent sample sets revealed decreased expression of WTAP in EC. We also found a decrease in overall m6A levels in EC, consistent with previous findings [44]. Furthermore, upon isolating ECSCs from the Ishikawa cell line, we observed that WTAP levels were lower in ECSCs than in Ishikawa cells, accompanied by a concurrent decrease in m6A levels. This led to the hypothesis that the reduction in WTAP and the resulting decrease in m6A modifications are crucial for sustaining the stemness of ECSCs. Subsequent experiments demonstrated that the reduction in WTAP promoted cell resistance, proliferation, invasion, metastasis, and self-renewal in ECCs and ECSCs. In most cases, m6A modification promotes the stability and expression of RNA [45], and the observed reduction in m6A due to decreased WTAP may lead to the downregulation of key genes essential for cellular self-regulation, thereby fostering the propagation and stemness of EC cells.

Subsequent transcriptome sequencing identified EGR1 as a downstream target gene of WTAP. RIP and MeRIP assays confirmed that WTAP can bind to and modulate the m6A modification status of EGR1 mRNA. Interestingly, WTAP did not influence the nascent RNA of EGR1 but significantly impacted the stability of its mRNA. Given the role of the IGF2BP family as m6A readers typically involved in RNA stabilization [36], further investigation into their interaction with EGR1 mRNA was undertaken. The findings indicated that IGF2BP3 serves as an m6A reader for EGR1, collaborating with WTAP to regulate EGR1 mRNA levels. This synergy suggests that the downregulation of WTAP, with subsequent effects on EGR1, might contribute to the increased stemness observed in ECSCs, underlining a complex regulatory network influencing EC pathogenesis and progression.

We further explored EGR1, and the results showed that EGR1 was significantly reduced in EC, and the reduction in EGR1 promoted cell resistance, proliferation, metastasis, invasion, and self-renewal while inhibiting apoptosis in ECCs and ECSCs. Previous studies have shown that EGR1, a tumor suppressor gene, plays a role in inhibiting tumor cell glycolysis in liver cancer [46]. EGR1 has also been shown to play a cancer-suppressing role in breast cancer [24]. In a recent study, EGR1 was also found to promote the antitumor activity of the chemotherapy drug doxorubicin [47]. A previous breast cancer study showed that EGR1 plays a role in cancer suppression and endocrine therapy by regulating PTEN, which also inhibits endometrial proliferation [48]. Therefore, we speculated that PTEN might be downstream of EGR1 in ECSCs, and the CHIP results indeed proved that EGR1 could transcribe PTEN.

PTEN is a classic tumor suppressor gene. We knocked down PTEN to promote cisplatin resistance; promote proliferation, migration, invasion, and self-renewal; and inhibit cell apoptosis in ECCs and ECSCs. In previous studies, reduced PTEN was found to promote the stemness of tumor stem cells in both ovarian and breast cancer [30, 49]. In this study, we demonstrated for the first time that WTAP reduction can reduce the stability of EGR1 mRNA by reducing the m6A modification of EGR1 mRNA and that EGR1 reduction reduces PTEN, which promotes the maintenance of ECSCs. Finally, our in vivo study showed that overexpression of WTAP, EGR1 and PTEN inhibited the growth of xenograft tumors, while overexpression of WTAP + EGR1 or EGR1 + PTEN further inhibited the growth of xenograft tumors. In addition, immunohistochemical experiments showed that overexpression of WTAP, EGR1 and PTEN inhibited the expression of Ki-67, NANOG and SOX2 in tumors in vivo, further demonstrating that inhibition of the WTAP/EGR1/PTEN axis promoted the progression of EC and the maintenance of stemness in ECSCs. It provides a new target for the treatment of EC.

## Conclusion

In conclusion, we discovered that WTAP downregulation in EC led to a decrease in the m6A modification of EGR1 mRNA. This reduction in m6A levels impaired the interaction between IGF2BP3 and EGR1 mRNA, subsequently diminishing the stability of EGR1 mRNA. The resulting reduction in EGR1 expression further decreased PTEN levels, disrupting the normal regulatory mechanisms and fostering malignant behaviors and stemness in ECSCs. This study elucidated the critical role of the WTAP/EGR1/PTEN axis, where its inhibition contributes to the loss of cellular regulation, thereby promoting EC malignancy and maintaining stem cell properties. Moreover, in vivo experiments reinforced the clinical relevance of the WTAP/EGR1/PTEN axis in EC therapy. Overexpression of this axis has good potential for attenuating EC progression, highlighting this axis as a promising therapeutic target for combating this disease. Collectively, our findings offer valuable insights into the molecular underpinnings of EC and suggest a novel therapeutic strategy to curb its progression by targeting the WTAP/EGR1/PTEN regulatory mechanism.

### Electronic supplementary material

Below is the link to the electronic supplementary material.

Supplementary Material 1

Supplementary Material 2

Supplementary Material 3

## Data Availability

The datasets used and/or analysed during the current study are available from the corresponding author on reasonable request.
