# Supplementary figures and images for: WTAP/IGF2BP3 mediated m6A modification of the EGR1/PTEN axis regulates the malignant phenotypes of endometrial cancer stem cells
Source: J Exp Clin Cancer Res. 2024 Jul 23;43:204. doi: 10.1186/s13046-024-03120-w (PMC11264439; doi:10.1186/s13046-024-03120-w)

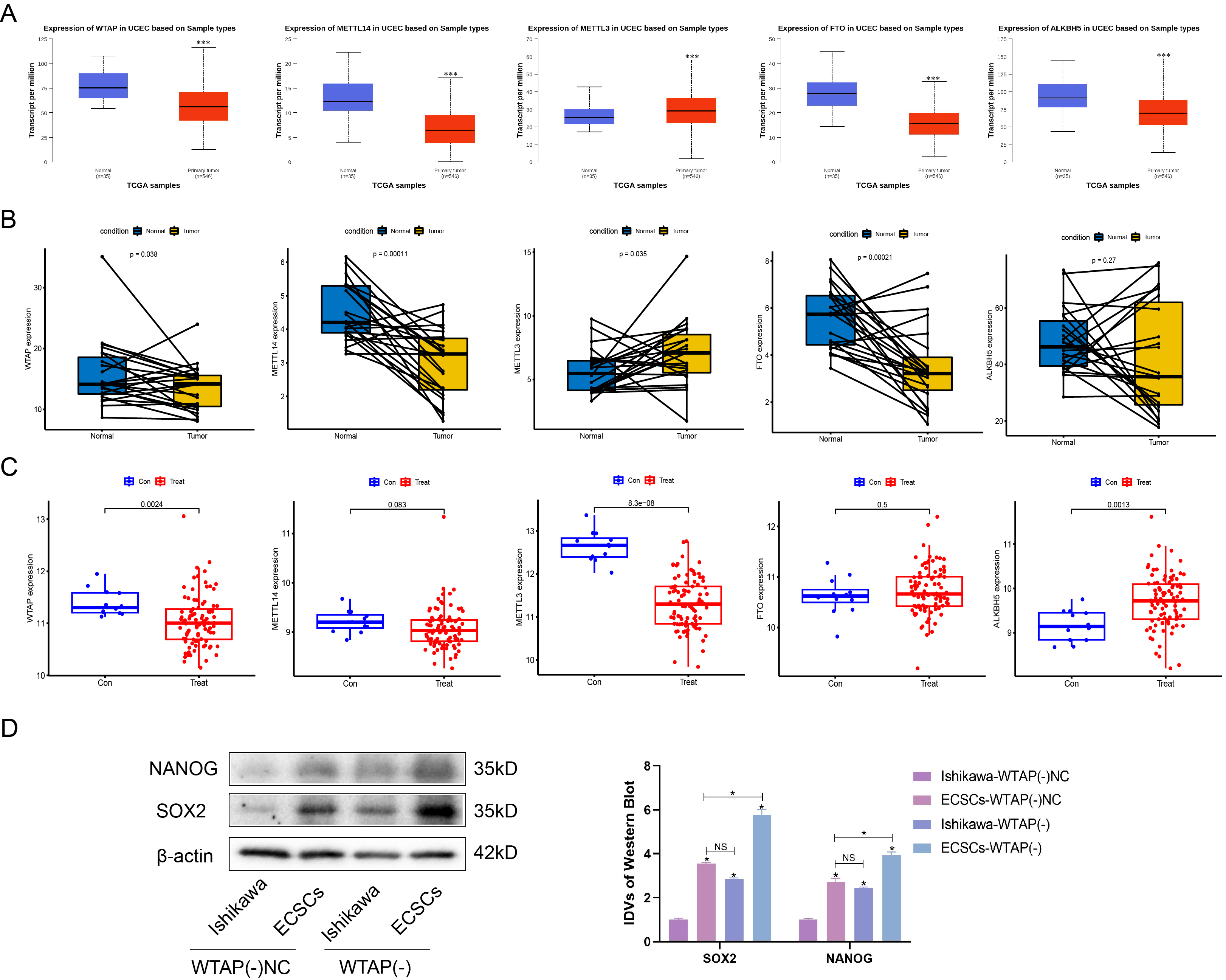

Supplement: Supplementary file 1 — Supplementary Material 1 [file 13046_2024_3120_MOESM1_ESM.jpg]

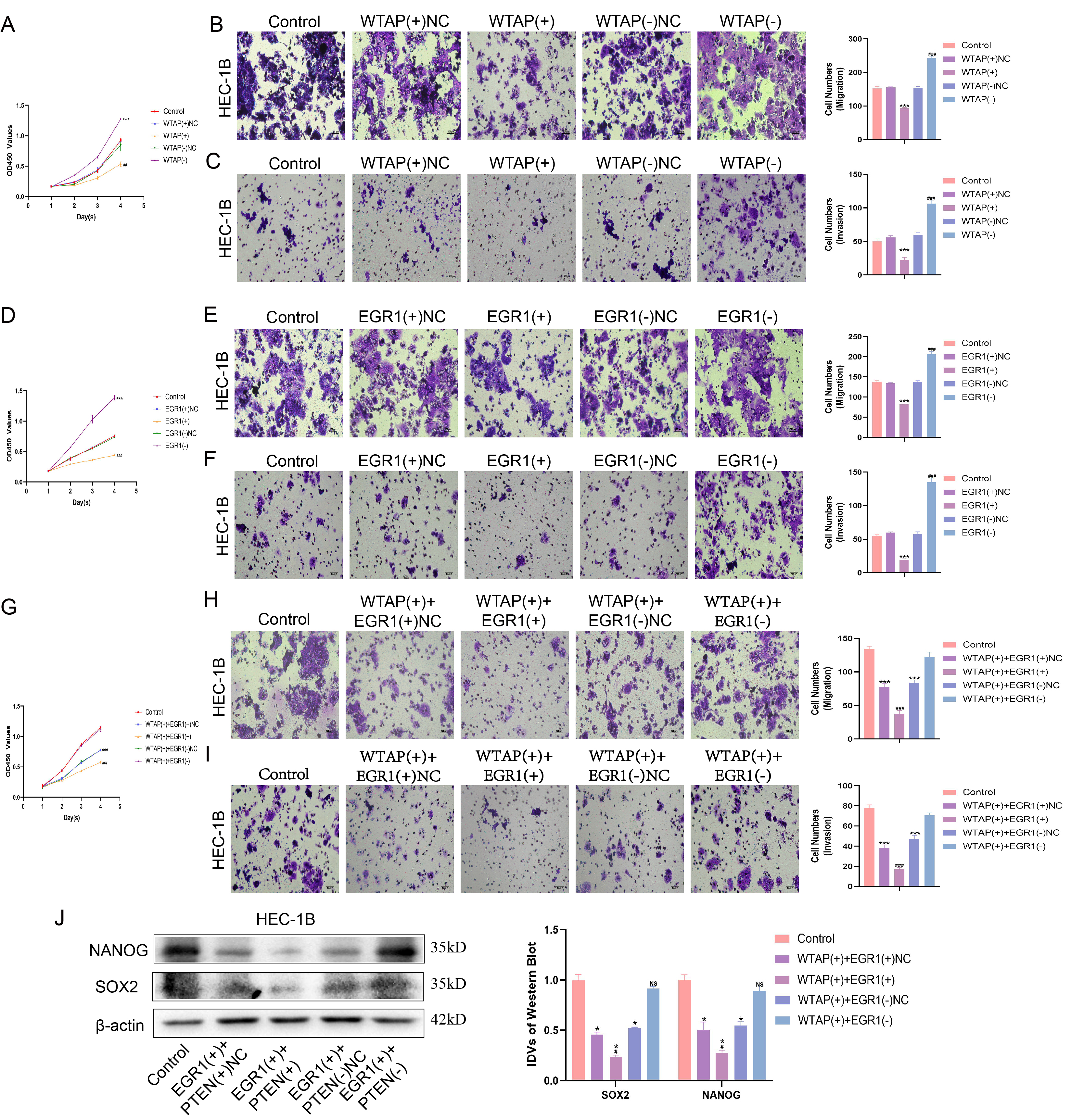

Supplement: Supplementary file 2 — Supplementary Material 2 [file 13046_2024_3120_MOESM2_ESM.jpg]

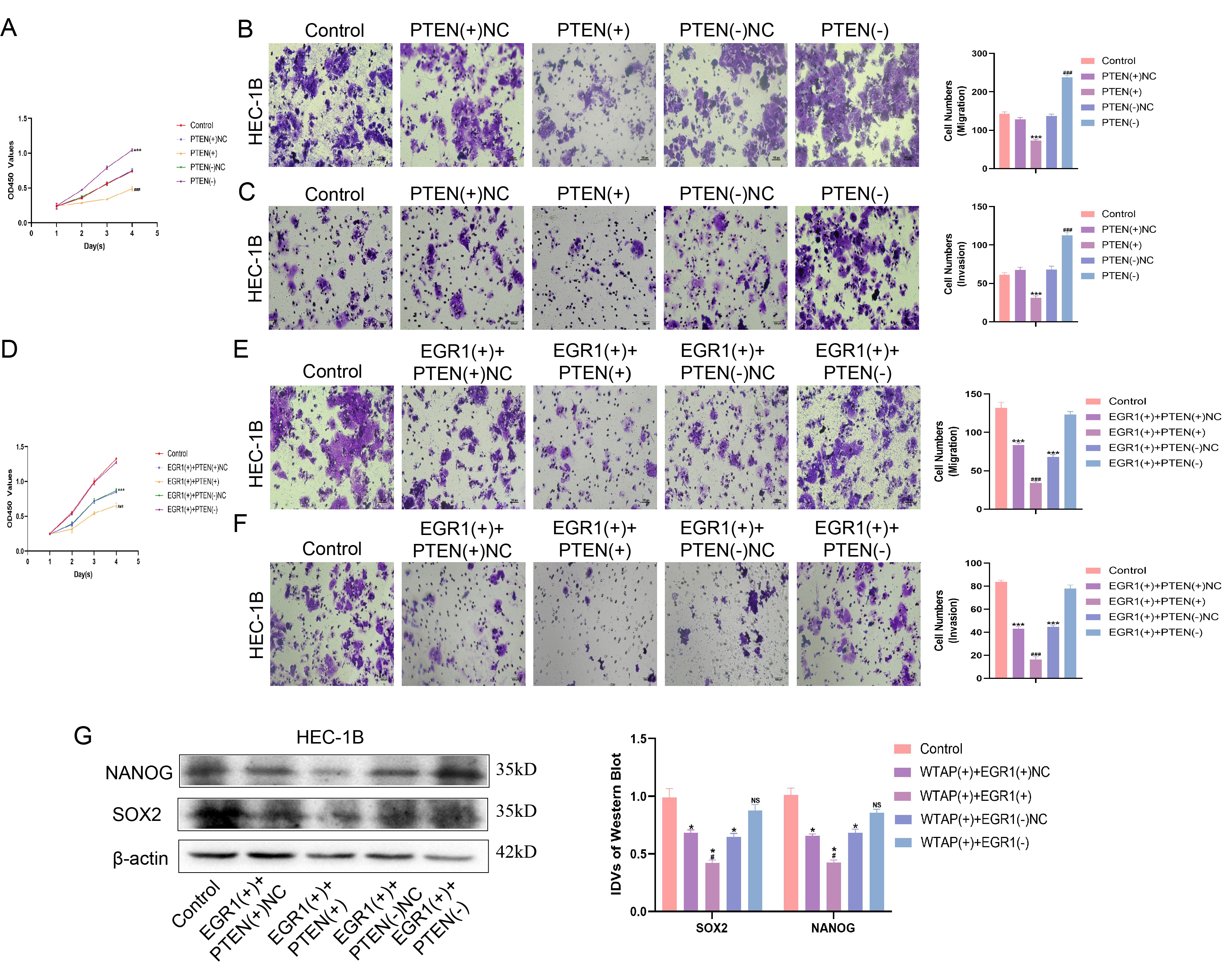

Supplement: Supplementary file 3 — Supplementary Material 3 [file 13046_2024_3120_MOESM3_ESM.jpg]
